# Sex differences in the rapid detection of neutral faces associated with emotional value

**DOI:** 10.1186/s13293-023-00567-y

**Published:** 2023-11-14

**Authors:** Akie Saito, Wataru Sato, Sakiko Yoshikawa

**Affiliations:** 1 Psychological Process Research Team, Guardian Robot Project, RIKEN, 2-2-2 Hikaridai, Seika-cho, Soraku-gun, Kyoto, 619-0288 Japan; 2 Field Science Education and Research Center, Kyoto University, Oiwake-cho, Kitashirakawa, Sakyo, Kyoto 606-8502 Japan; 3 Faculty of the Art and Design, Kyoto University of The Arts, 2-116 Uryuuzan, Kitashirakawa, Sakyo, Kyoto 606-8501 Japan

**Keywords:** Visual search, Learning, Neutral faces, Emotional value, Sex differences

## Abstract

**Background:**

Rapid detection of faces with emotional meaning is essential for understanding the emotions of others, possibly promoting successful interpersonal relationships. Although few studies have examined sex differences in the ability to detect emotional faces, it remains unclear whether faces with emotional meaning capture the attention of females and males differently, because emotional faces have visual saliency that modulates visual attention. To overcome this issue, we tested the rapid detection of the neutral faces associated with and without learned emotional value, which are all regarded as free from visual saliency. We examined sex differences in the rapid detection of the neutral female and male faces associated with emotional value.

**Methods:**

First, young adult female and male participants completed an associative learning task in which neutral faces were associated with either monetary rewards, monetary punishments, or no monetary outcomes, such that the neutral faces acquired positive, negative, and no emotional value, respectively. Then, they engaged in a visual search task in which previously learned neutral faces were presented as discrepant faces among newly presented neutral distractor faces. During the visual search task, the participants were required to rapidly identify discrepant faces.

**Results:**

Female and male participants exhibited comparable learning abilities. The visual search results demonstrated that female participants achieved rapid detection of neutral faces associated with emotional value irrespective of the sex of the faces presented, whereas male participants showed this ability only for male faces.

**Conclusions:**

Our results demonstrated that sex differences in the ability to rapidly detect neutral faces with emotional value were modulated by the sex of those faces. The results suggest greater sensitivity to faces with emotional significance in females, which might enrich interpersonal communication, regardless of sex.

**Supplementary Information:**

The online version contains supplementary material available at 10.1186/s13293-023-00567-y.

## Plain language summary

Speedy detection of faces with emotional meaning plays a fundamental role in social interactions. However, it is unclear whether females and males differ in their ability to rapidly detect neutral faces associated with newly acquired emotional meaning/value. This study examined the sex differences in the rapid detection of neutral female and male faces associated with emotional value subsequent to associative learning. During learning, neutral faces were paired with monetary reward or punishment, such that they acquired positive or negative emotional value, respectively. In a subsequent visual search task, previously learned neutral faces were presented as discrepant faces among newly presented neutral faces, and the participants had to rapidly identify the discrepant faces. The results showed that, among female participants, neutral faces associated with reward and punishment were detected more rapidly than neutral faces not associated with monetary outcomes, irrespective of the sex of the face stimuli. By contrast, male participants only showed the rapid detection of neutral male faces. The results suggest enhanced sensitivity to faces with emotional meaning among females, which is consistent with the notion of greater sensitivity to emotional/social information in females.

**Supplementary Information:**

The online version contains supplementary material available at 10.1186/s13293-023-00567-y.

## Highlights

- Female and male participants exhibited a comparable level of successful associative learning.
- Female participants showed rapid detection of neutral faces associated with emotional value, irrespective of the sex of those faces.
- Male participants showed rapid detection of neutral male faces with emotional value only.
- Male participants did not show a detection advantage for neutral female faces with emotional value.

**Supplementary Information:**

The online version contains supplementary material available at 10.1186/s13293-023-00567-y.

## Background

Faces with emotional meaning are rapidly detected, which is considered an essential part of emotion processing in interpersonal relationships. Similar to the case of emotional facial expressions, such as angry or smiling faces, neutral faces with emotional meaning/significance acquired via learning have been proven to be rapidly detected in prior studies [1]. In that study, participants learned associations between inherently neutral faces and monetary rewards, punishments, or non-monetary outcomes via associative learning. The participants subsequently showed more rapid detection of the value-associated neutral faces compared to the faces with non-monetary outcomes in a visual search task. It has been suggested that acquired emotional meaning/significance enhances attention and promotes the detection of value-associated neutral stimuli (i.e., faces [2–4]) In this sense, shared mechanisms are assumed to underlie the efficient search for value-associated neutral and emotional facial expressions [5, 6].

Several studies have reported that the processing of facial emotional information, such as the ability to recognize emotional facial expressions, could be modulated by sex differences [7–10]. For example, female participants reportedly responded faster than male participants in a labeling task involving a variety of emotional facial expressions[10], and exhibited stronger attention-related electric brain potentials in response to emotional facial expressions[11]. Moreover, female participants in several studies were more sensitive to emotional social signals [12–14]. Another interesting phenomenon relevant to sex differences in emotion processing is that there is an *own sex bias*, whereby people are better able to recognize or detect facial stimuli of the same sex [8, 9, 15, 16]. These studies indicated that the own sex bias is less evident in female than male participants. In short, the evidence suggests female superiority in the processing of facial emotional information.

In contrast, few studies have examined sex differences in the rapid detection of emotional facial expressions [16, 17], and the results have been inconsistent. Specifically, the studies tested the detection speed of emotional facial expressions using a visual search paradigm and found female superiority for some emotional facial expressions in one study [16], but no sex differences in another study [17]. To the things more complicated, emotional facial expressions possess not only emotional meaning but also entail visual saliency (e.g., oblique eyebrows displayed on angry faces) [18], and it has been known that visual saliency modulates visual attention [19]. It, therefore, remains unclear as to the issue of sex differences in the rapid detection of faces with emotional meaning.

To examine sex differences in the rapid detection of faces with emotional meaning, it might be beneficial to use neutral faces that have acquired emotional value, because, unlike emotional facial expressions, they are not confounded by visual saliency. To the best of our knowledge, no studies to date have examined sex differences in the rapid detection of neutral faces associated with emotional value, and it is unclear whether sex differences would occur in the rapid detection of neutral faces associated with emotional value.

Examining sex differences in the rapid detection of neutral faces with emotional value might help promote our understanding of psychiatric disorders. It has been demonstrated that psychiatric disorders related to the reward system manifest differently between the sexes [20]. For instance, females show higher prevalence rates of depression than males [21, 22], and addictions and the outcomes of addiction emerge differently by biological sex and gender [23]. Given that these disorders arise from, in part, decreased or enhanced visual attention toward reward-related stimuli, sex differences in detection efficiency for value-associated stimuli may partly explain the sex differences in prevalence rates of psychiatric disorders.

In this study, we aimed to examine whether there are sex differences in the rapid detection of neutral faces associated with monetary rewards and punishments, compared to neutral faces with no monetary outcomes in a visual search task, after successfully learning reward contingencies. We also examined whether the sex of the neutral face (female or male) modulated any such sex differences in the rapid detection of value-associated neutral faces by manipulating the sex of the neutral face as an additional experimental factor.

**Fig. 1:**
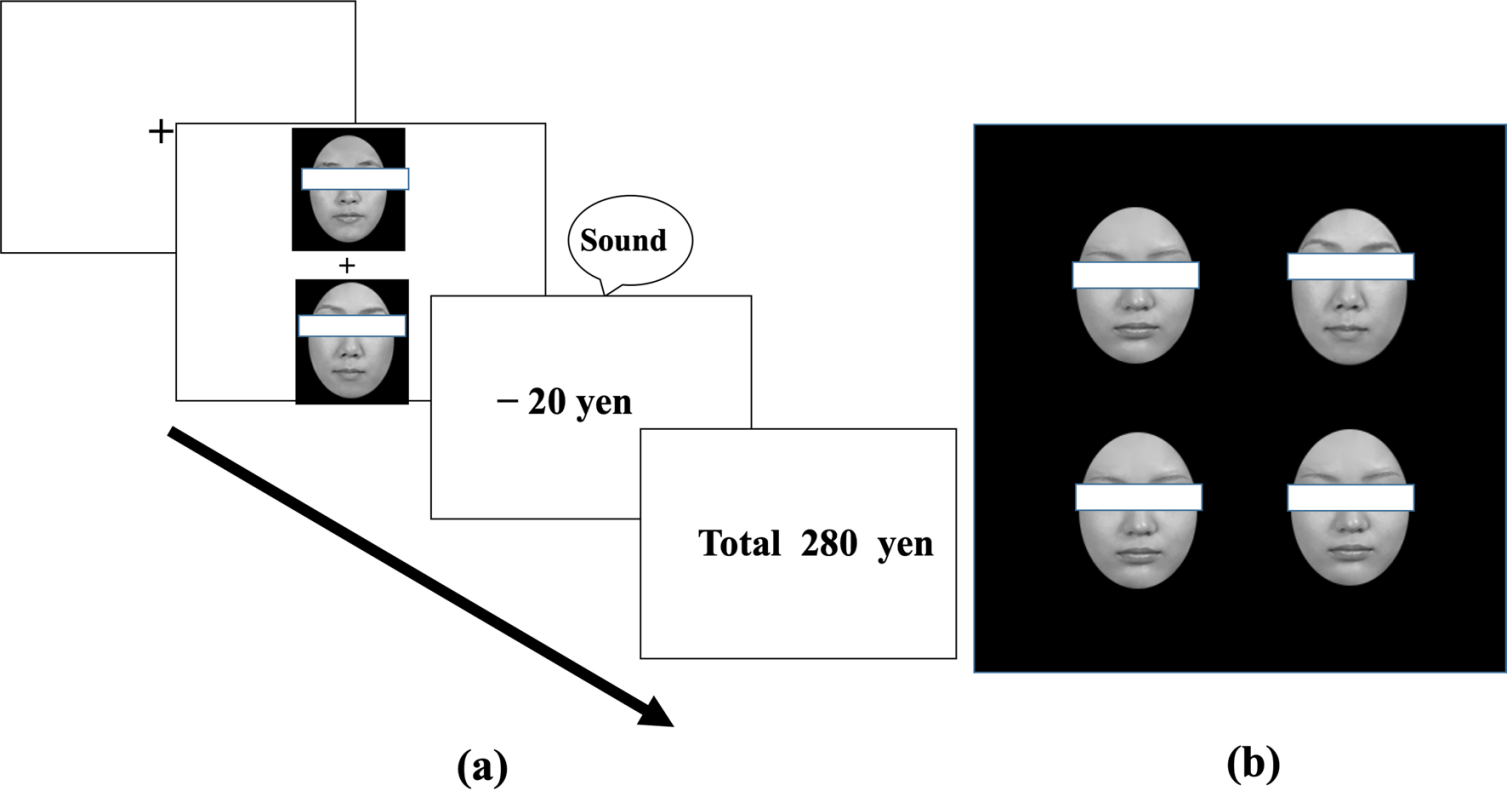
**a**, **b** Example trials in the learning task (**a)** and the visual search task (**b**). In the learning task, participants were required to choose one face from each pair to maximize their earnings. In the visual search task, the participants had to identify one discrepant face embedded among distractor faces. A representative search trial in the target-present condition (for neutral female stimuli). In the actual experiment, the target faces were not covered with eye masks

Given the superior emotion processing and sensitivity to the emotional faces of females [10, 11, 13, 14], we hypothesized that female participants would show rapid detection of neutral faces associated with emotional value irrespective of those faces’ sex, more advantageously compared to their male counterparts. This hypothesis was tested using an associative learning task implemented in previous value-learning studies[1, 24–26] and a subsequent visual search task [1] (Fig. 1). In the learning task, participants were required to choose one face from a pair of neutral faces to maximize their earnings for each experimental trial (300 trials in total). Each of the three types of pairs was assigned to either monetary gain (reward), monetary loss (punishment), or a zero-outcome condition. After learning had been established, the participants engaged in the subsequent visual search task, in which previously learned neutral faces were presented as targets (discrepant) faces among newly presented identical neutral distractor faces, and the participants had to rapidly detect discrepant faces embedded among several identical distractor faces.

## Methods

### Participants

Seventy-seven young adults participated in the experiment (38 females and 39 males; mean ± *SD* age = 22.2 ± 2.0 years overall), including the data of 29 young adults who took part in a previous study using the same experimental procedure [1]. All participants were Japanese undergraduate or graduate students with normal or corrected-to-normal vision. The sample size was determined based on an a priori power analysis using G*Power software (ver.3.1.9.2; [27]). We assumed a 2 × 2 × 3 mixed-design analysis of variance (ANOVA) as an approximation of linear mixed effects model analysis. We set an *α* of 0.05, power (1–*β*) of 0.80, effect size *f* of 0.25 (medium size), and correlation among repeated measures of 0.5. The results showed that more than 40 participants were needed. Participants were paid 2,500 Japanese yen for completing the 120-min session, in addition to a predetermined bonus of 1,000 Japanese yen in the associative learning task. This study had a three-factor mixed design, with participant sex (female or male) and face stimuli sex (female or male) as between-participant factors and with the type of emotional value (reward [monetary gain], punishment [monetary loss], and zero [no outcome]) as within-participant factors. Categorizing the sex of the participants was based on self-reports and physical appearance. Five participants were excluded on the grounds of possible visual impairment, procedural errors, or difficulty categorizing them into either sex group. Thus, the final sample included 72 participants (35 females and 37 males; mean ± *SD* age = 22.1 ± 1.7 years). The cohorts’ demographic data according to sex are presented in Additional file 1: Table S1. Participants provided written informed consent. All experimental procedures were approved by the Ethics Committee of the Unit for Advanced Studies of the Human Mind, Kyoto University, and were performed in accordance with the institutional ethical guidelines and the Declaration of Helsinki.

### Apparatus

Stimuli were displayed on a 19-inch monitor (HM903D-A; Iiyama, Tokyo, Japan), with a refresh rate of 150 Hz and a resolution of 1024 × 768 pixels. Stimuli were controlled by Presentation 14.9 software (Neurobehavioral Systems, San Francisco, CA, USA) running on a Windows computer (HP Z200 SFF; Hewlett-Packard, Tokyo, Japan). Participants’ responses were collected by a response box (RB-530; Cedrus, San Pedro, CA, USA) with a 2–3-ms response time (RT) resolution.

### Stimuli

For the main experiment, 6 grayscale photographs of neutral faces of each sex (12 faces in total) were used as targets, along with one distractor neutral face of each sex. The stimuli were taken from a database of faces of Japanese faces [28]. Each photograph was adjusted for light and shade using Photoshop 5.0 (Adobe, San Jose, CA, USA), and the mean luminance of the stimuli was equalized using MATLAB R2017b (MathWorks, Natick, MA). The stimuli were also controlled for attractiveness and distinctiveness. Each stimulus was cropped and embedded within an ellipsoid frame to exclude distinctive factors (e.g., hairstyle, facial contours), with subtended visual angles of 3.5° horizontally and 4.5° vertically. Preliminary experiments confirmed that detection speed did not significantly differ among target faces in the visual search task (*F*(5,35) = 2.18, *p* = 0.079 for male faces; *F*(5,30) = 1.05, *p* = 0.41 for female faces). We also administered an additional preliminary experiment to determine whether stimulus sex (i.e., the female or male target faces used for the main experiment) could be accurately discriminated by young adults using an online experiment (N = 20, females = 10, mean ± *SD,* 31.8 ± 3.7, males = 10 mean ± *SD,* 31.8 ± 2.7). The results showed that the participants’ ability to discriminate the sex of the faces presented was significantly higher than the chance level (mean (*SE*) = 0.93(0.01), *t* (19) = 32.77, *p* < 0.001 by one-sample *t* test against 0.5). There was no significant difference in the recognition accuracy of the sex of the faces between female and male participants (mean (*SE*) = 0.94 (0.02) for female participants, mean (*SE*) = 0.93 (0.02) for male participants, *t* (18) = 0.26, *p* = 0.80 by paired *t* tests), indicating that both groups were able to equally and accurately discriminate the sex of the faces.

*Associative learning task.* There were three pairs of neutral faces for each sex. Approximately half (*n* = 35) of the participants were shown neutral female faces (35 participants), and the remaining participants (*n* = 37) were shown neutral male faces. We attempted to equalize the number of male and female participants for the two types of face stimuli conditions. In each type of face stimuli condition, one pair was allocated to one of the three value-type conditions (reward, punishment, or zero outcomes). The face pairs were fixed throughout the task, so that each face invariably appeared with its partner in each value condition. The allocation of pairs to value conditions was counterbalanced across the participants. In the reward and punishment conditions, one face of each pair was designated as the target; the choice of the target resulted in a monetary reward (20 yen increase in each trial) in the reward condition and a monetary loss (20 yen decrease in each trial) in the punishment condition, with a probability of 80% (otherwise 20% probability of zero outcomes). For the nontarget face in each pair, the contingency was reversed (20% probability of monetary reward or loss, and otherwise 80% probability of zero outcomes). In the zero-outcome condition, one face was assigned to a target, but no monetary outcomes were provided (always zero outcomes), regardless of whether the participants selected the target or non-target face. Across the participants, target face allocations in each condition were counterbalanced. Each participant completed the learning task, experiencing either of the 24 patterns of combinations for the assigned group.

*Visual search task*. Three target faces from the value conditions to which individual participants were exposed during the learning task, along with one neutral distractor face, were presented to participants. In each face stimuli conditions, the distractor face was selected from the database mentioned above using the same criteria applied for the selection of the six faces in the learning task. In this task, four faces appeared simultaneously in a square configuration (11.0° × 11.0°) on the search display, with each face appearing in either of four positions separated by 40°. One target face, along with three identical neutral distractor faces, appeared in either of the four positions in an imaginary square configuration for the target-present trials. Each target face appeared an equal number of times in the four positions (a total of 32 appearances per target). For the target-absent trials, the four faces presented were all identical distractor faces.

### Procedure

The participants were seated in a chair 80 cm from the monitor screen, with the chin in a fixed position, in a dimly lit soundproofed room (Science Cabin, Takahashi Kensetsu, Tokyo, Japan). The participants were administered the associative learning and visual search experiments as part of a larger study involving other cognitive tasks and questionnaires.

*Associative learning task.* Participants were instructed to take part in a betting game and to select a face from each pair of faces that appeared on the computer screen according to their “gut feeling.” Faces were selected by pressing the corresponding button on the response box. They were told that the goal was to maximize their earnings. They were also informed that the earned money would be paid after the experiment and were encouraged to do their best to earn money. Because the participants were not informed about which face in each pair was the target face, they had to learn the contingencies. In each trial, a pair of faces appeared in the center of the screen after a 0.9° × 0.9° fixation cross was presented for 500 ms. One face appeared 2.5° above the fixation cross and the other appeared 2.5° below the fixation cross. Each face appeared in the two positions in pseudorandom order. After the participant made a selection, a “price” message appeared on the screen (+ 20 yen, − 20 yen, or 0 yen), accompanied by a sound that indicated whether the answer was correct or incorrect (no sound for the “0 yen” message). Then, the running total of yen earned was displayed for 1800 ms. Each pair of faces appeared on the screen 10 times per block (a total of 30 trials), and the main experiment consisted of 10 blocks of 30 trials (300 total trials). To prevent the consecutive presentation of identical face pairs in the same positions within each block, the order of presentation of each pair of faces was pseudorandomized. Task instructions and 30 practice trials preceded the main experiment.

*Visual search task*. Participants were informed that there would be no monetary reward or loss in this task. In each trial, a fixation cross was shown for 500 ms on the screen, followed by the simultaneous presentation of a stimulus array of four faces. The participants were given instructions to indicate whether a discrepant face appeared among the four faces, or whether faces were the same, by pressing the corresponding button on the response box as quickly and accurately as possible. The response button allocations were counterbalanced across the participants. Each block contained target-present trials (8 each for reward, loss, and zero-outcome conditions) and 24 target-absent trials, and the main experiment consisted of four blocks of 48 trials (192 trials in total). To prevent the consecutive presentation of identical targets in the same positions, the trials’ order of presentation was pseudorandomized within each block. Twenty-four practice trials preceded the main experimental trials.

After the experiment, debriefing took place. The participants were asked to report anything they had noticed while performing the two tasks, such as whether they had recognized that the discrepant (target faces) in the visual search task were the same as those that had appeared in the previous associative learning task. Almost all participants (54/56 participants) reported that they had not noticed that the previously learned faces were presented as targets in the subsequent visual search task (i.e., frequent answers were those that they had found the task easy to perform and were able to identify discrepant faces based on facial features).

### Data analyses

Data were analyzed using SPSS (ver.10.0J; SPSS Japan, Tokyo, Japan) and MATLAB 2020a (MathWorks, Natick, MA, USA). The *α* level was set at 0.05.

*Associative learning task.* A three-way analysis of variance (ANOVA) with the value type (reward [monetary gain], punishment [monetary loss], or zero [no outcome]) as a within-participant factor and participant sex (female or male) and stimulus sex (female or male) as between-participant factors was conducted to examine whether successful learning rates differed between the male and female participants. Follow-up multiple comparisons were performed using the Ryan method. The effect size indices (i.e., *η*^2^_*p*_) were reported for the ANOVA but not for the follow-up analyses due to adjustment difficulty [29]. To compare the mean proportion of target face choices between block 10 and block 1 in each value condition, paired *t *tests were performed (two-tailed).

*Visual search task*. For each participant, the mean RTs of the correct responses for each condition of the target-present trials were calculated after excluding responses longer than 3 s and the measurement ± 2 *SD* from the mean. Regarding accuracy data, three-way ANOVA of the mean accuracy of each participant with value type as a within-participant factor and participant sex and stimulus sex as between-participant factors was conducted. Regarding RT data, a series of linear mixed effects model analyses were performed to analyze the RTs. Fixed-effect independent variables included participant sex (female or male), stimulus sex (female or male), value type (reward [monetary gain], punishment [monetary loss], or zero [no outcome]), and their two-way and three-way interactions. Random by-participant intercepts were added as per standard ANOVA; besides, model comparison using Akaike Information Criteria suggested that the model with only by-participant intercepts was preferred compared with that with by-participant intercepts and slopes. In cases with a significant three-way interaction, follow-up simple–simple effects analyses for value type (reward vs. no-outcome and punishment vs. no-outcome) were conducted for each participant sex × stimulus sex condition. When there were significant higher order interactions, other main effects and interactions were not subjected to interpretation, because they were deemed likely to be qualified by the higher order interactions. Standardized coefficients (*β*; in absolute value) were reported as the effect size indices [30].

## Results

### Associative learning task

**Fig. 2:**
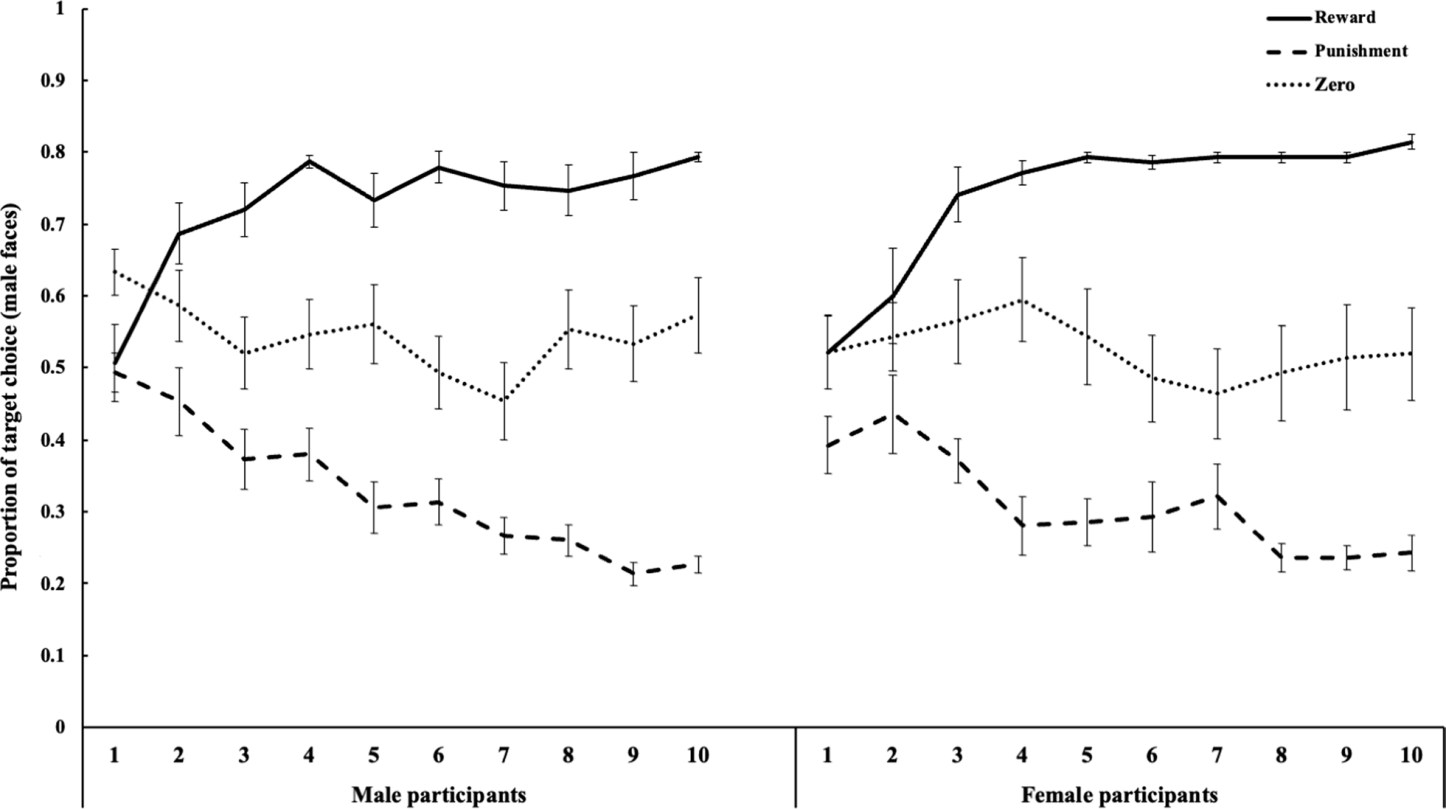
Mean (± standard error) proportion of male target faces selected during each block in the reward, punishment, and zero-outcome conditions

**Fig. 3:**
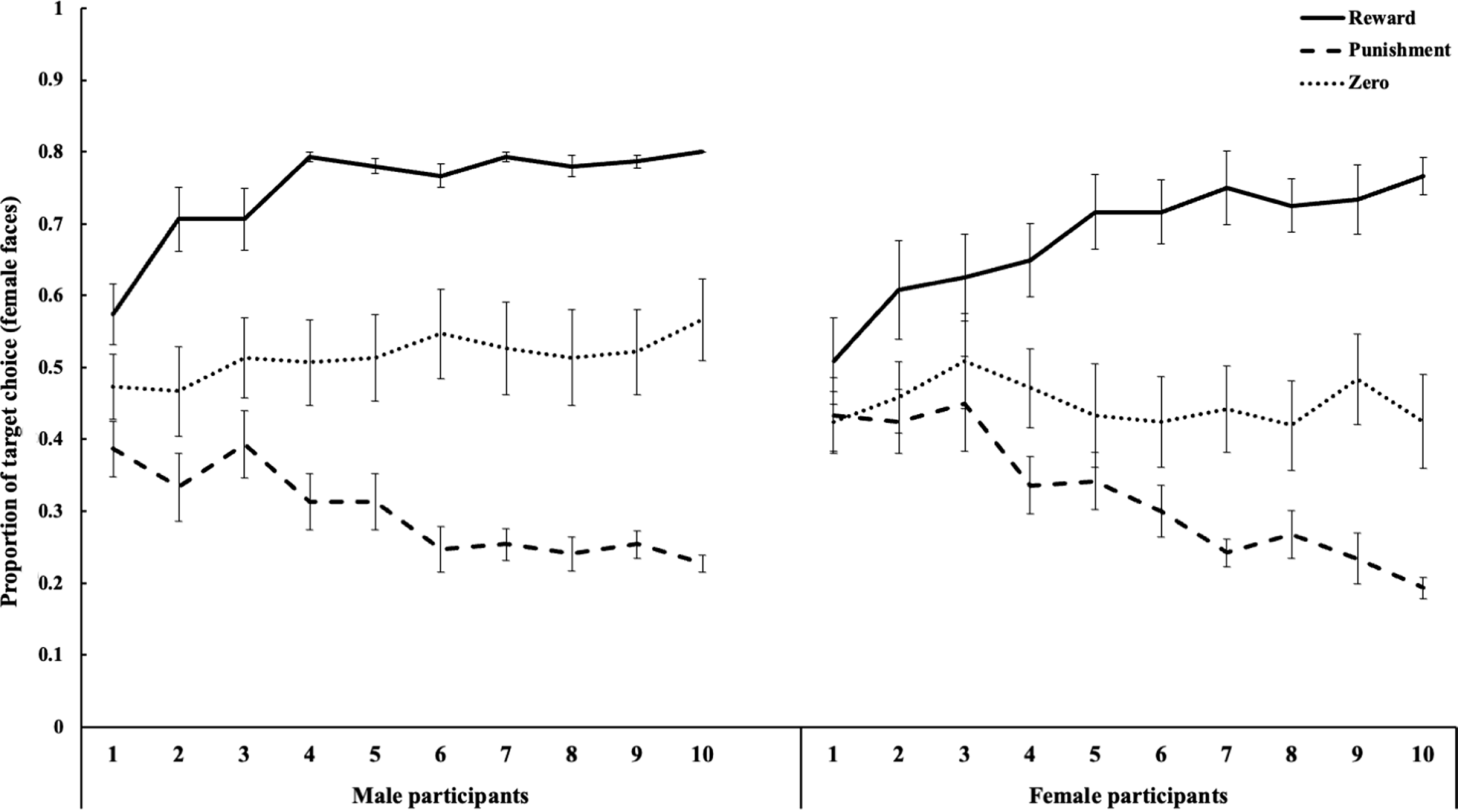
Mean (± standard error) proportion of female target faces selected during each block in the reward, punishment, and zero-outcome conditions

An optimal face selection accuracy rate > 65% in the final block (i.e., targets and non-targets in the reward and he punishment conditions, respectively) was considered indicative of successful learning, following previous studies [1, 2]. There were 26 and 30 successful female and male learners, respectively. The proportion of target face selections during the final block (10 block) among the successful learners was analyzed with a three-way ANOVA with participant sex, stimulus sex, and value type as factors. Only the main effect of value type (*F*(1,52) = 251.66, *p* < 0.001, *η*^2^_*p*_ = 0.83) was significant. The three-way interaction was not significant (*F*(2,104) = 0.08,* p* = 0.923, *η*^2^_*p*_ = 0.002) and no other significant effects were observed (*F* < 2.97, *p* > 0.091, *η*^2^_*p*_ < 0.054), indicating no sex differences in the ability to learn the emotional value associations. Multiple comparisons using the Ryan method showed that target selection rates in the reward and punishment conditions differed significantly from that in the zero-outcome condition (*t*(104) = 10.74, *p* < 0.001 for the reward vs. zero condition,* t*(104) = 11.78, *p* < 0.001, for the punishment vs. zero condition). Differences in the target selection rates between the reward and punishment conditions were also significant (*t*(104) = 22.52, *p* < 0.001). The results indicate that, irrespective of their sex, the participants were more likely to choose target faces with reward and to avoid target faces with punishment, compared to target faces without value during the final block of the learning task. A similar pattern of successful learning was shown by comparing the selection rates of the target faces between the first and final blocks (Figs. 2 and 3). The results revealed that the selection of target faces increased significantly in the reward condition, decreased in the punishment condition, and did not change in the zero-outcome condition regardless of stimulus sex or participant sex (*t* (11) = -3.74, *p* = 0.003, *d* = 1.08 for reward, *t* (11) = 4.27, *p* = 0.001, *d* = 1.23 for punishment, and *t* (11) = 0.00, *p* = 1.000, *d* = 0.00 for zero-outcome female faces among female participants; *t* (14) = -5.41, *p* < 0.001, *d* = 1.40 for reward, *t* (14) = 4.13, *p* = 0.001, *d* = 1.07 for punishment, and *t* (14) = −1.56, *p* = 0.140, *d* = 0.40 for zero-outcome female faces among male participants; *t* (13) = -5.43, *p* < 0.001, *d* = 1.45 for reward, *t* (13) = 2.67, *p* = 0.019, *d* = 0.71 for punishment, and *t* (13) = 0.06, *p* = 0.953, *d* = 0.02 for zero-outcome male faces among female participants; *t* (14) = -5.47, *p* < 0.001, *d* = 1.41 for reward, *t* (14) = 8.00, *p* < 0.001, *d* = 2.07 for punishment, and *t* (14) = 1.01, *p* = 0.328, *d* = 0.26 for zero-outcome male faces among male participants).

### Visual search task

Data from the participants who demonstrated successful learning in the previous associative learning task were analyzed.

*Accuracy data.* Face detection accuracy was high for all the value conditions for both face types among female and male participants (see Additional file 1: Table S2 and Additional file 1: Fig. S1). We conducted an analysis of the mean accuracy of each participant using participant sex × stimulus sex × value type ANOVA, and found no significant three-way interaction (*F* (2,104) = 2.64, *p* = 0.076, *η*^2^_*p*_ = 0.048), or no other significant effects (*F*(2,104) < 0.40,* p* > 0.67, *η*^2^_*p*_ < 0.008), except the significant main effects of participant sex (*F* (1,52) = 3.62, *p* = 0.030, *η*^2^_*p*_ = 0.07; female participants performed better than male participants) and value type (*F* (1,52) = 4.14, *p* = 0.047, *η*^2^_*p*_ = 0.07). Multiple comparisons for the value type conditions using the Ryan method demonstrated that accuracy in the punishment condition was significantly higher than in the zero-outcome condition (*t*(104) = 2.70, *p* = 0.008). The differences between the reward and zero-outcome conditions or between the reward and punishment conditions were not significant(*t*(104) = 1.32, *p* = 0.188; *t*(104) = 1.38, *p* = 0.171). Because there were no significant higher order interactions in terms of accuracy in the visual search task, the results indicate no evident speed–accuracy trade-off phenomenon.

**Fig. 4:**
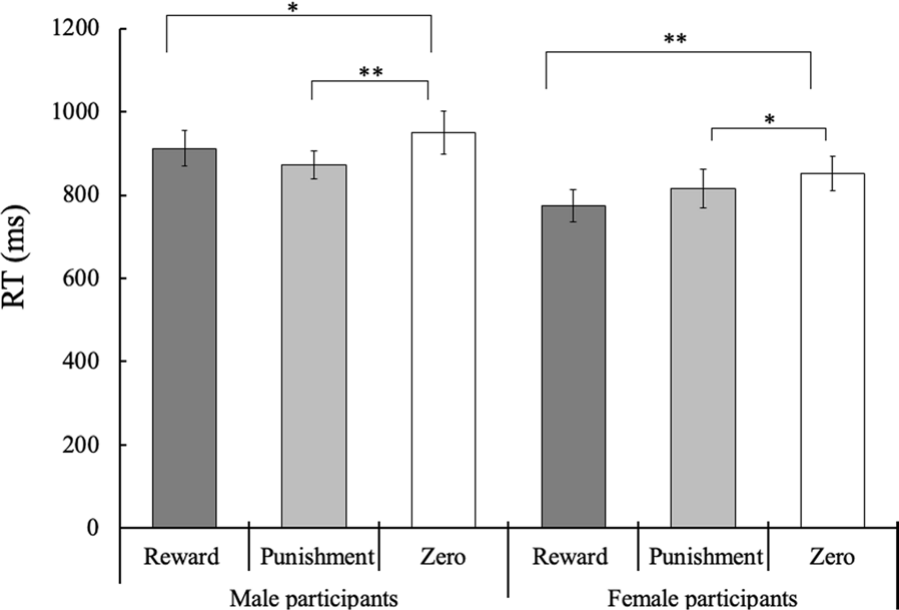
Mean (± standard error) reaction times (RTs) of the male and female groups for the detection of neutral male faces associated with reward, punishment, and zero outcomes in the visual search task. Asterisks denote significant differences between conditions (**: *p* < 0.01; *: *p* < 0.05)

**Fig. 5:**
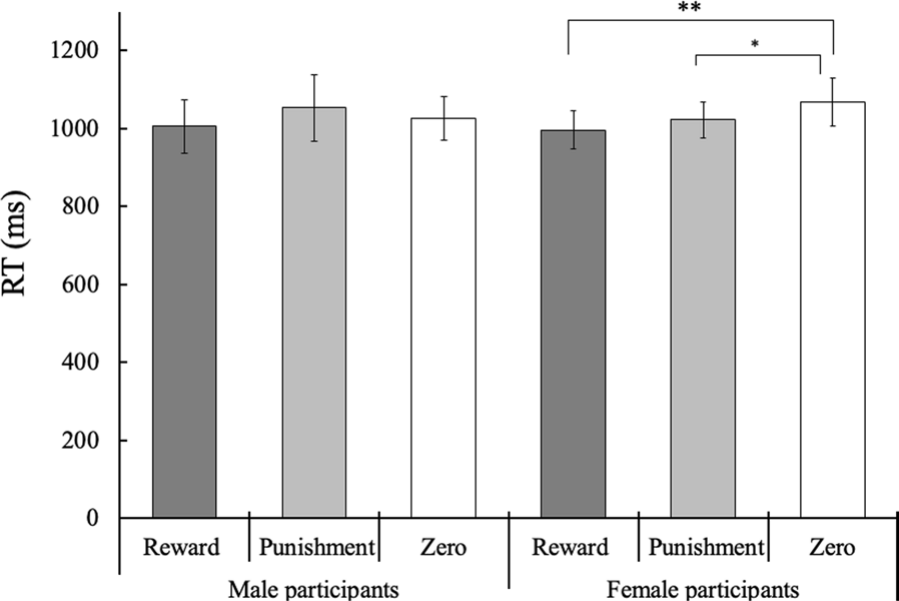
Mean (± standard error) reaction times (RTs) of the male and female groups for the detection of neutral female faces associated with reward, punishment, and zero outcomes in the visual search task. Asterisks denote significant differences between conditions (**: *p* < 0.01; *: *p* < 0.05)

*RT data*. The mean RTs for each target condition in each group are shown in Figs. 4 and 5. Linear mixed effects model analysis revealed a significant three-way interaction among participant sex, stimulus sex, and value type (*F*(2,4703.1) = 5.64, *p* = 0.004, *β* = 0.02). Besides, the two-way interaction between stimulus sex and value type (*F*(2, 4703.10) = 3.23, *p* = 0.039, *β* = 0.01) and main effects of stimulus sex and value type (*F*(1, 55.704) = 10.86, *p* = 0.002, *β* = 0.26; *F*(2, 4703.1) = 18.12, *p* < 0.001, *β* = 0.05, respectively) were significant.

To interpret the three-way interaction, follow-up analyses were performed to examine the effect of the value type (reward vs. zero outcome and punishment vs. zero outcome) for each participant sex × stimulus sex condition. For female participants, there were significant differences between the reward and zero outcome conditions for both stimulus sex conditions (*F*(1,666.25) = 11.84, *p* < 0.001 for female stimuli and *F*(1,781.16) = 24.58, *p* < 0.001 for male stimuli), as well as between the punishment and zero-outcome conditions (*F*(1,683.93) = 4.51, *p* = 0.034 for female stimuli and *F*(1,774.15) = 5.59, *p* = 0.018 for male stimuli). The results indicated that for female participants, faces associated with reward and punishment were detected more rapidly than those associated with zero outcomes, which were irrelevant to the sex of the faces displayed.

In contrast, male participants detected value-associated faces significantly more rapidly than zero-outcome faces only for male face stimuli (*F*(1,820.12) = 5.81, *p* = 0.016 for reward vs. zero-outcome and *F*(1,825.24) = 22.02, *p* < 0.001 for punishment vs. zero-outcome). For female face stimuli, there were no significant differences between the reward and zero-outcome conditions (*F*(1,822.97) = 1.72, *p* = 0.189) or between the punishment and zero-outcome conditions (*F*(1,821.92) = 0.69, *p* = 0.406). These results indicated that male participants showed a detection advantage only for same-sex value-associated target faces.

## Discussion

This study focused on examining sex differences in the ability to rapidly detect neutral faces associated with monetary rewards and punishment in a visual search task after reward contingencies were acquired. We also examined whether the rapid detection of neutral faces with emotional value depends on the sex of the stimuli (faces).

Analyses of learning performance indicated no sex differences in the ability to learn emotional value associations, and irrespective of their sex, the participants were more likely to choose reward-associated neutral faces and avoid punishment-associated neutral faces than neutral faces without emotional value during the final block of the learning task. Moreover, the likelihood of choosing reward-associated neutral faces and punishment-associated neutral faces increased or decreases as learning became established, while the likelihood of choosing target neutral faces in the zero-outcome condition did not change, for participants of either sex. These results suggest that participants were able to acquire positive or negative emotional value throughout the learning task and that there are no sex differences in this behavioral pattern of learning, which is consistent with prior studies reporting no sex differences in behavioral patterns (i.e., accuracy) in reward- and punishment-based learning [31–34].

More importantly, the results of the visual search task demonstrated sex differences in the rapid detection of reward- and punishment-associated neutral faces, modulated by the sex of the face stimuli. Although female participants exhibited a detection advantage for both reward- and punishment-associated neutral face stimuli irrespective of the sex of the faces, male participants showed a detection advantage for male faces only. These results suggest that emotional value acquired via learning facilitates the rapid detection of value-associated neutral faces (reflected in shorter RTs in the value-associated condition than in the no-monetary outcome condition) by females and by males for same-sex faces, which aligns with previous studies showing that neutral faces acquired emotional value via associative learning [2, 3, 5] and that there is a relationship between subjective emotional ratings and rapid detection of value-associated neutral faces [1].

Male participants did not show a detection advantage for the neutral faces associated with emotional value when the discrepant neutral faces in the visual search task were female faces. It is plausible that the acquired emotional value did not exert sufficient power to facilitate the rapid detection of value-associated neutral faces of the opposite sex (female faces) among male participants. In this sense, it might be said that the female participants were better able to rapidly detect value-associated neutral faces than the male participants. Female participants’ superior performance in the rapid detection of value-associated neutral faces was also shown by comparing the accuracy with which the female and male participants detected the value-associated neutral faces.

The detection advantage of the value-associated neutral faces of our female participants accords with previous studies indicating enhanced visual attention to emotional faces (non-threat-related faces) and reward- and punishment-related stimuli among female participants [10, 11, 13, 16, 35]. It has been documented that females are more likely than males to be aware of and pay attention to other people’s emotions and to pick up social signals to facilitate communication and increase social bonding [9, 36] This probably might be attributable to combined influences of the evolutionarily and socially/culturally determined role of females as primary caregivers [37–39], whereby females are expected to take the lead in nurturing their children, regardless of children’s sex [9, 40]. Our results suggest that such a sex/gender-based-specific pattern of attention allocation to emotionally significant stimuli among females occurs even when they view neutral faces with newly learned emotional value.

Male participants were vulnerable to modulations by the sex of the neutral faces, only showing rapid detection of value-associated neutral faces of the same sex (own sex bias), which is consistent with prior studies reporting a greater effect of own sex bias in the processing of emotional stimuli among male than female participants [8, 9, 15, 16] for threat-related faces. Possibly, the male participants might have paid more attention to the value-associated same-sex faces than to the same-sex faces without value relative to the case of opposite-sex faces. Similar to the hypothetical accounts applied to female participants, this pattern of results might reflect the evolutionarily and socially/culturally constrained roles of males, where rapidly extracting information about the emotions of other males in competitive situations would have been critical for survival from an evolutionary perspective (i.e., inter-male competition [41], and might also be crucial for participating in economic and political life in today’s society.

Alternatively, our male participants might have placed the same amount of value on the zero-outcome neutral female faces as they did on the neutral female faces associated with reward and punishment, which might have led to the absence of the rapid detection of value-associated female neutral faces among male participants. Relevant prior functional neuroimaging studies have reported increased activation of the amygdala among male participants but not female participants during exposure to neutral faces of the opposite sex vs. the same sex [8, 15]. Interpreting the result patterns, Fisher et al.[15] have argued that sex differences in mate selection preferences [42] might explain why females are more likely than males to seek a mate based on nonphysical characteristics, whereas physical attractiveness is reportedly a necessity to males for seeking their mate [42, 43]. Given this possibility, it is likely the physical attractiveness of the female–neutral faces in our study might have affected the detection performance for our male participants; even female–neutral faces without monetary value might have been as attractive as the value-associated female–neutral faces for the male participants.

Significant implications of the superior performance in the rapid detection of value-associated neutral faces of our female participants concern the relationship between their enhanced attention to value-associated neutral faces and emotional well-being. Our results suggest that young female adults pay considerable attention to both opposite- and same-sex faces with emotional value. Although enhanced awareness and attention to faces with emotional meaning might be adaptive in terms of understanding the emotions of others and thus facilitate communication in most cases, it can also be a “double-edged sword.”: it has been documented that among young females, interpersonal problems are particularly associated with psychological distress [21]. It might be the case that greater attention to the faces of emotionally significant others (i.e., loved ones) drives an excessive focus on interpersonal relationships, possibly leading to the development of mental illnesses due to interpersonal problems among young females, as proposed in prior studies reporting a greater sensitivity of females to emotional stimuli, including stimuli related to reward and punishment [11, 35, 44]. It is of note, however, that for most adult females, greater attention to and engagement with emotions facilitates adaptive emotion regulation [45], which may, in turn, promote well-being. Against this background, our findings might imply that some vulnerable female adults may be prone to mental problems if they have greater sensitivity to emotional faces.

It is also notable that although the own sex bias exhibited by our male participants in the rapid detection of value-associated neutral faces appears adaptive in today’s society, social/cultural norms inevitably change as the members of the society change. Undifferentiated attention to female faces irrespective of behavioral/emotional significance by males may not be as adaptive in the future as it is considered to be at present. Paying enhanced attention to the facial expressions of behaviorally/emotionally significant females, to better understand their emotions, may ultimately improve males’ own well-being.

In addition, our results might offer insight into the biological mechanisms underlying the sex differences in the rapid detection of neutral faces with emotional value. First, regarding the neural substrates, prior studies have demonstrated that the amygdala plays a key role in the processing of emotional faces [46–51]. Some neuropsychological studies further showed that amygdala lesion impair the rapid detection of emotional facial expressions [47, 48, 51], indicating the amygdala activity underlies the rapid detection of emotional facial expressions. Crucially, neuroscientific study has revealed commonality in the amygdala between the processing of innate and learned emotional stimuli [52, 53]. This suggests that the shared underlying mechanisms in the amygdala between innate and learned emotional stimuli. Considering these findings, it might be possible to assume that the amygdala could be involved in the rapid detection of neutral faces with acquired emotional value, and our participants' pattern of RTs (an indicator of detection speed) might likely reflect the amygdala activity in the brain. Regarding the relevance of our study to the biology of sex differences, some research has shown that amygdala activity is more lateralized in male participants than in female participants during the viewing of emotional facial expressions [54, 55], but see [56]. Bilateral activation in females is proposed to contribute to their superior processing of emotional facial expression [57–59]. Together with these data, our results suggest that sex differences in amygdala activity may underlie the sex differences in the rapid detection of neutral faces with learned emotional value observed in this study.

Second, regarding sex differences in hormonal influences on social/emotional behavior, oxytocin has been demonstrated to modulate emotion processing [60–62]. Crucially, oxytocin reportedly facilitates early stages of emotional facial expression processing [63–65] but see [66], suggesting a relationship between oxytocin and the rapid detection of emotional facial expressions. Regarding sex differences in endogenous oxytocin, female participants reportedly have higher plasma oxytocin levels than male participants [67, 68], which might explain the superior performance of processing emotional facial expressions in female participants, [10, 11, 13, 14] given the relationship between oxytocin and emotion processing. Combined with the idea that the shared mechanisms underlie the efficient search for value-associated neutral and emotional facial expressions [5, 6], differences in oxytocin levels may also underlie sex differences in the rapid detection of neutral faces with learned emotional value.

There are several limitations that should be considered. First, the sample of our study was Japanese adults. Our results might have been affected by the gender role expectations prevalent in Japanese society; Japan has a low ranking for gender equality (Global Gender Gap Index [69]). Therefore, the generalizability of our results might be limited. Future studies should clarify whether the sex/gender differences in the ability to rapidly detect value-associated neutral faces observed in our young Japanese participants generalize to young adults living in other countries with greater gender equality, such as Northern European countries. Second, we did not provide a detailed discussion of the influence of sex differences in brain structure/functions or hormonal/chromosomal status on the sex-specific patterns of rapid detection of value-associated neutral faces; such factors inarguably modulate the processing of affective stimuli differently depending on participants’ sex [9, 70]. Further research should examine how and to what extent evolutionary and social/cultural factors interact with biological/genetic ones to generate sex/gender differences in the ability to rapidly detect faces with emotional value.

In conclusion, the present study demonstrated that sex differences in the ability to rapidly detect neutral faces with emotional value differed according to the sex of the face stimuli. These results suggest that sex/gender-specific characteristics in the processing of emotional stimuli, which are thought to be influenced by both evolutionary and social–cultural actors, also exert power on the rapid detection of newly learned neutral faces with emotional value.

## Perspectives and significance

Our finding suggests that, whether learned or innate, emotion modulates the ways of perception and behavioral output in a different manner between female and male adults.

### Supplementary Information

**Additional file 1: Table S1.** Demographic data. **Table S2.** Mean (with *SE*) correct proportions of each type of target face detection in the visual search task among female and male participants who succeeded in the previous associative learning task. **Figure S1.** Mean (with *SE*) correct proportions of target face detection in the visual search task among female and male participants who succeeded in the previous associative learning task, collapsing the factor of stimulus sex.

## Data Availability

Data sets are available from the corresponding author upon reasonable request.

## References

[1] Saito A, Sato W, Yoshikawa S (2022). Rapid detection of neutral faces associated with emotional value. Cogn Emot.

[2] Gupta R, Hur YJ, Lavie N (2016). Distracted by pleasure: effects of positive versus negative valence on emotional capture under load. Emotion.

[3] Hammerschmidt W, Sennhenn-Reulen H, Schacht A (2017). Associated motivational salience impacts early sensory processing of human faces. Neuroimage.

[4] Wentura D, Müller P, Rothermund K (2014). Attentional capture by evaluative stimuli: gain- and loss-connoting colors boost the additional-singleton effect. Psychon Bull Rev.

[5] Bliss-Moreau E, Barrett LF, Wright CI (2008). Individual differences in learning the affective value of others under minimal conditions. Emotion.

[6] Fujiwara J, Tobler PN, Taira M, Iijima T, Tsutsui KI (2009). Segregated and integrated coding of reward and punishment in the cingulate cortex. J Neurophysiol.

[7] Connolly HL, Lefevre CE, Young AW, Lewis GJ (2019). Sex Differences in emotion recognition: evidence for a small overall female superiority on facial disgust. Emotion.

[8] Kret ME, Pichon S, Grèzes J, de Gelder B (2011). Men fear other men most: gender specific brain activations in perceiving threat from dynamic faces and bodies-an fMRI study. Front Psychol.

[9] Kret ME, De Gelder B (2012). A review on sex differences in processing emotional signals. Neuropsychologia.

[10] Saylik R, Raman E, Szameitat AJ (2018). Sex differences in emotion recognition and working memory tasks. Front Psychol.

[11] Pfabigan DM, Lamplmayr-Kragl E, Pintzinger NM, Sailer U, Tran US (2014). Sex differences in event-related potentials and attentional biases to emotional facial stimuli. Front Psychol.

[12] Baron-Cohen S, Wheelwright S (2004). The empathy quotient: an investigation of adults with asperger syndrome or high functioning autism, and normal sex differences. J Autism Dev Disord.

[13] Proverbio AM, Zani A, Adorni R (2008). Neural markers of a greater female responsiveness to social stimuli. BMC Neurosci.

[14] Rattel JA, Mauss IB, Liedlgruber M, Wilhelm FH (2020). Sex differences in emotional concordance. Biol Psychol.

[15] Fischer H, Sandblom J, Herlitz A, Fransson P, Wright CI, Bäckman L (2004). Sex-differential brain activation during exposure to female and male faces. NeuroReport.

[16] Williams MA, Mattingley JB (2006). Do angry men get noticed?. Curr Biol.

[17] Sawada R, Sato W, Uono S, Kochiyama T, Toichi M (2014). Sex differences in the rapid detection of emotional facial expressions. PLoS ONE.

[18] Calvo MG, Nummenmaa L (2008). Detection of emotional faces: Salient physical features guide effective visual search. J Exp Psychol Gen.

[19] Theeuwes J (1992). Perceptual selectivity for color and form. Percept Psychophys.

[20] Becker JB, McClellan ML, Reed BG (2017). Sex differences, gender and addiction. J Neurosci Res.

[21] Leach LS, Christensen H, Mackinnon AJ, Windsor TD, Butterworth P (2008). Gender differences in depression and anxiety across the adult lifespan: The role of psychosocial mediators. Soc Psychiatry Psychiatr Epidemiol.

[22] Sikes-Keilp C, Rubinow DR (2021). In search of sex-related mediators of affective illness. Biol Sex Differ.

[23] Becker JB, McClellan M, Reed BG (2016). Sociocultural context for sex differences in addiction. Addict Biol.

[24] Anderson BA, Laurent PA, Yantis S (2011). Learned value magnifies salience-based attentional capture. PLoS ONE.

[25] Chen N, Wei P (2019). Reward association alters brain responses to emotional stimuli: ERP evidence. Int J Psychophysiol.

[26] Raymond JE, O’Brien JL (2009). Selective visual attention and motivation. Psychol Sci.

[27] Faul F (2007). G*Power 3: a flexible statistical power analysis program for the social, behavioral, and biomedical sciences. Behav Res Methods.

[28] Sato W, Hyniewska S, Minemoto K, Yoshikawa S (2019). Facial expressions of basic emotions in Japanese laypeople. Front Psychol.

[29] Sánchez MET, Cervantes RJM (2016). Generalized eta squared for multiple comparisons on between-groups designs. Psicothema.

[30] Lorah J (2018). Effect size measures for multilevel models: definition, interpretation, and TIMSS example. Large-scale Assess Educ.

[31] Dhingra I, Zhang S, Zhornitsky S, Wang W, Le TM, Li CSR (2021). Sex differences in neural responses to reward and the influences of individual reward and punishment sensitivity. BMC Neurosci.

[32] Greimel E, Bakos S, Landes I, Töllner T, Bartling J, Kohls G (2018). Sex differences in the neural underpinnings of social and monetary incentive processing during adolescence. Cogn Affect Behav Neurosci.

[33] Barman A, Richter S, Soch J, Deibele A, Richter A, Assmann A (2014). Gender-specific modulation of neural mechanisms underlying social reward processing by Autism Quotient. Soc Cogn Affect Neurosci.

[34] Warthen KG, Boyse-Peacor A, Jones KG, Sanford B, Love TM, Mickey BJ (2020). Sex differences in the human reward system: Convergent behavioral, autonomic and neural evidence. Soc Cogn Affect Neurosci.

[35] Dumais KM, Chernyak S, Nickerson LD, Janes AC (2018). Sex differences in default mode and dorsal attention network engagement. PLoS ONE.

[36] Brody LR, Hall JA, Lewis M, Haviland-Jones JM, Barrett LF (2008). Gender and emotion in context. Handbook of emotions 3.

[37] Babchuk WA, Hames RB, Thompson RA (1985). Sex differences in the recognition of infant facial expressions of emotion: The primary caretaker hypothesis. Ethol Sociobiol.

[38] Kuhn SL, Stiner MC (2006). What’s a mother to do? The division of labor among Neandertals and modern humans in Eurasia. Curr Anthropol.

[39] Proverbio AM (2023). Sex differences in the social brain and in social cognition. J Neurosci Res.

[40] Fischer AH, Rodriguez Mosquera PM, Van Vianen AEM, Manstead ASR (2004). Gender and culture differences in emotion. Emotion.

[41] Archer J (2019). The reality and evolutionary significance of human psychological sex differences. Biol Rev.

[42] Feingold A (1992). Gender differences in mate selection preferences: a test of the parental investment model. Psychol Bull.

[43] Li NP, Kenrick DT, Bailey JM, Linsenmeier JAW (2002). The necessities and luxuries of mate preferences: testing the tradeoffs. J Pers Soc Psychol.

[44] Legget KT, Cornier MA, Bessesen DH, Mohl B, Thomas EA, Tregellas JR (2018). Greater reward-related neuronal response to hedonic foods in women compared with men. Obesity.

[45] Nolen-Hoeksema S (2012). Emotion regulation and psychopathology: the role of gender. Annu Rev Clin Psychol.

[46] Adolphs R, Tranel D, Damasio H, Damasio A (1994). Impaired recognition of emotion in facial expressions following bilateral damage to the human amygdala. Nature.

[47] Bach DR, Hurlemann R, Dolan RJ (2015). Impaired threat prioritisation after selective bilateral amygdala lesions. Cortex.

[48] Domínguez-Borràs J, Moyne M, Saj A, Guex R, Vuilleumier P (2020). Impaired emotional biases in visual attention after bilateral amygdala lesion. Neuropsychologia.

[49] Fitzgerald DA, Angstadt M, Jelsone LM, Nathan PJ, Phan KL (2006). Beyond threat: amygdala reactivity across multiple expressions of facial affect. Neuroimage.

[50] Morris JS, Frith CD, Perrett DI, Rowland D, Young AW, Calder AJ, Dolan RJ (1996). A differential neural response in the human amygdala to fearful and happy facial expressions. Nature.

[51] Sato W, Usui N, Sawada R, Kondo A, Toichi M, Inoue Y (2021). Impairment of emotional expression detection after unilateral medial temporal structure resection. Sci Rep.

[52] Khalil V, Faress I, Mermet-Joret N, Kerwin P, Yonehara K, Nabavi S (2023). Subcortico-amygdala pathway processes innate and learned threats. Elife.

[53] Gore F, Schwartz EC, Brangers BC, Aladi S, Stujenske JM, Likhtik E (2015). Neural representations of unconditioned stimuli in basolateral amygdala mediate innate and learned responses. Cell.

[54] Killgore WDS, Yurgelun-Todd DA (2001). Sex differences in amygdala activation during the perception of facial affect. NeuroReport.

[55] Schneider S, Peters J, Bromberg U, Brassen S, Menz MM, Miedl SF (2011). Boys do it the right way: Sex-dependent amygdala lateralization during face processing in adolescents. Neuroimage.

[56] Sergerie K, Chochol C, Armony JL (2008). The role of the amygdala in emotional processing: a quantitative meta-analysis of functional neuroimaging studies. Neurosci Biobehav Rev.

[57] Proverbio AM, Riva F, Martin E, Zani A (2010). Neural markers of opposite-sex bias in face processing. Front Psychol.

[58] Lazar SM, Evans DW, Myers SM, Moreno-De Luca A, Moore GJ (2014). Social cognition and neural substrates of face perception: Implications for neurodevelopmental and neuropsychiatric disorders. Behav Brain Res.

[59] Pavlova MA (2017). Sex and gender affect the social brain: beyond simplicity. J Neurosci Res.

[60] Lischke A, Berger C, Prehn K, Heinrichs M, Herpertz SC, Domes G (2012). Intranasal oxytocin enhances emotion recognition from dynamic facial expressions and leaves eye-gaze unaffected. Psychoneuroendocrinology.

[61] Di Simplicio M, Harmer CJ (2016). Oxytocin and emotion processing. J Psychopharmacol.

[62] Van Ijzendoorn MH, Bakermans-Kranenburg MJ (2012). A sniff of trust: meta-analysis of the effects of intranasal oxytocin administration on face recognition, trust to in-group, and trust to out-group. Psychoneuroendocrinology.

[63] Domes G, Sibold M, Schulze L, Lischke A, Herpertz SC, Heinrichs M (2013). Intranasal oxytocin increases covert attention to positive social cues. Psychol Med.

[64] Hugrass L, Labuschagne I, Price A, Crewther DP, Author C. Intranasal oxytocin modulates very early visual processing of emotional faces. BioRxiv, 2021: 2021–04.

[65] Tillman R, Gordon I, Naples A, Rolison M, Leckman JF, Feldman R (2019). Oxytocin enhances the neural efficiency of social perception. Front Hum Neurosci.

[66] Guastella AJ, Carson DS, Dadds MR, Mitchell PB, Cox RE (2009). Does oxytocin influence the early detection of angry and happy faces?. Psychoneuroendocrinology.

[67] Marazziti D, Baroni S, Mucci F, Piccinni A, Moroni I, Giannaccini G (2019). Sex-related differences in plasma oxytocin levels in humans. Clin Pract Epidemiol Ment.

[68] Plasencia G, Luedicke JM, Nazarloo HP, Carter CS, Ebner NC (2019). Plasma oxytocin and vasopressin levels in young and older men and women: functional relationships with attachment and cognition. Psychoneuroendocrinology.

[69] Global gender gap index, 2023 World Economic Forum. 2023.

[70] Cahill L, Aswad D (2015). Sex influences on the brain: an issue whose time has come. Neuron.

