# Supplementary material for: Sex differences in the rapid detection of neutral faces associated with emotional value
Source: Biol Sex Differ. 2023 Nov 14;14:84. doi: 10.1186/s13293-023-00567-y (PMC10644416; doi:10.1186/s13293-023-00567-y)
Supplement: Supplementary file 1 — Additional file 1: Table S1. Demographic data. Table S2. Mean (with SE) correct proportions of each type of target face detection in the visual search task among female and male participants who succeeded in the previous associative learning task. Figure S1. Mean (with SE) correct proportions of target face detection in the visual search task among female and male participants who succeeded in the previous associative learning task, collapsing the factor of stimulus sex. [file 13293_2023_567_MOESM1_ESM.doc]

**Additional file 1**

Table S1. Demographic data.

|  | Female participants | Male participants |
| --- | --- | --- |
| Age  Years of Education | 22.1(1.8)  15.6(1.6) | 22.0(1.7)  15.5(1.6) |

Table S2. Mean (with *SE*) correct proportions of each type of target face detection in the visual search task among female and male participants who succeeded in the previous associative learning task.

|  | Female target face | |  | Male target face | |  |
| --- | --- | --- | --- | --- | --- | --- |
| Participant sex | reward | punishment | zero | reward | punishment | zero |
| Female | 0.95(0.02) | 0.98(0) | 0.94(0.02) | 0.97(0.01) | 0.97(0.01) | 0.96(0.01) |
| Male | 0.94(0.02) | 0.93(0.02) | 0.93(0.02) | 0.94(0.03) | 0.95(0.01) | 0.91(0.03) |

**
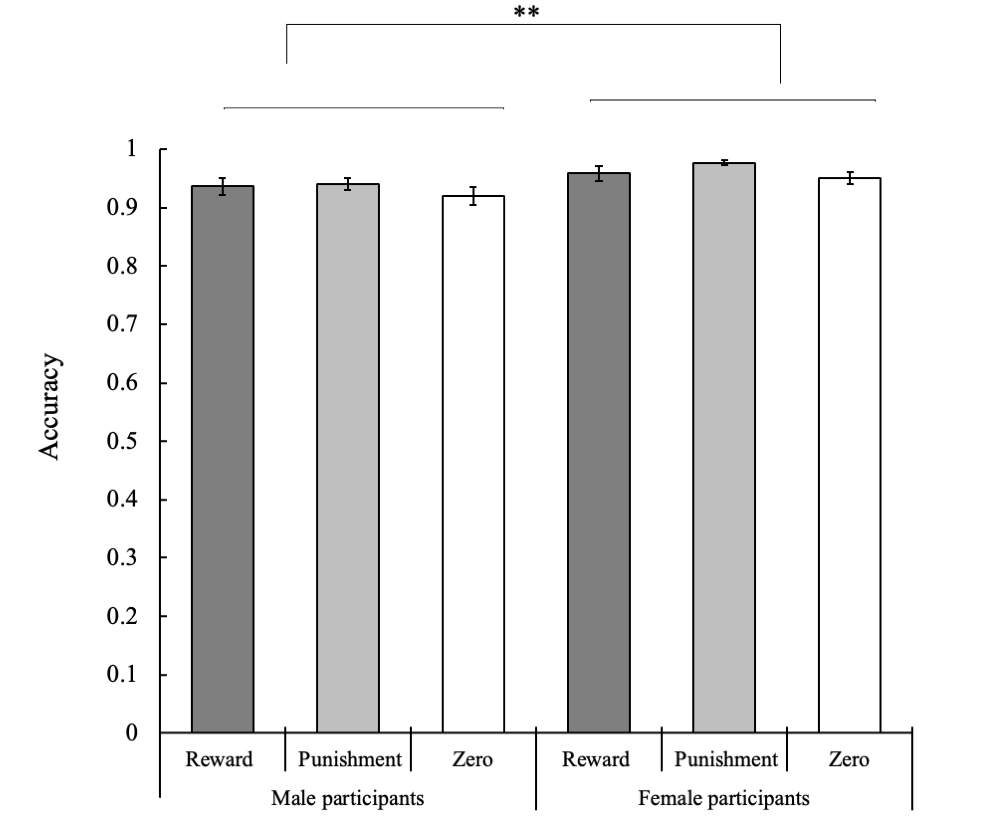
**

Figure S1 Mean (with *SE*) correct proportions of target face detection in the visual search task among female and male participants who succeeded in the previous associative learning task, collapsing the factor of stimulus sex.

Additional data

Performance on the associative learning during block 1 and block 10 (proportions of target choice in each value condition). Categories of stimulus sex and participant sex were between-participant factors. Learning success indicates whether the participant in this number met the criterion of successful learners in this task.

|  |  |  |  |  | Block 1 |  |  | Block 10 | |  |
| --- | --- | --- | --- | --- | --- | --- | --- | --- | --- | --- |
| Participant No. | Stimulus sex | Participant Sex (0=female, 1=male) | Learning success = 1 Unsuccess = 0) | Age | Reward target | Punishment target | Zero target | Reward target | Punishment target | Zero target |
| 1 | male | 0 | 1 | 21 | 0.80 | 0.30 | 0.70 | 0.80 | 0.20 | 0.70 |
| 2 | male | 0 | 1 | 23 | 0.80 | 0.30 | 0.70 | 0.80 | 0.30 | 0.70 |
| 3 | male | 1 | 1 | 22 | 0.60 | 0.50 | 0.40 | 0.80 | 0.20 | 0.60 |
| 4 | male | 1 | 1 | 23 | 0.30 | 0.60 | 0.50 | 0.70 | 0.20 | 0.50 |
| 5 | male | 0 | 1 | 23 | 0.40 | 0.40 | 0.50 | 0.80 | 0.20 | 0.40 |
| 6 | male | 1 | 1 | 23 | 0.70 | 0.30 | 0.60 | 0.80 | 0.20 | 0.80 |
| 7 | male | 0 | 1 | 22 | 0.30 | 0.30 | 0.40 | 0.80 | 0.20 | 0.60 |
| 8 | male | 0 | 1 | 20 | 0.20 | 0.70 | 0.30 | 0.80 | 0.20 | 0.20 |
| 9 | male | 1 | 1 | 25 | 0.70 | 0.40 | 0.70 | 0.80 | 0.30 | 0.60 |
| 10 | male | 1 | 1 | 21 | 0.70 | 0.40 | 0.70 | 0.80 | 0.20 | 0.80 |
| 11 | male | 1 | 1 | 20 | 0.40 | 0.50 | 0.60 | 0.80 | 0.20 | 0.70 |
| 12 | male | 0 | 1 | 21 | 0.80 | 0.30 | 0.80 | 0.80 | 0.20 | 0.80 |
| 13 | male | 1 | 1 | 27 | 0.60 | 0.60 | 0.60 | 0.80 | 0.20 | 0.80 |
| 14 | male | 1 | 1 | 23 | 0.40 | 0.50 | 0.50 | 0.80 | 0.20 | 0.20 |
| 15 | male | 1 | 1 | 22 | 0.80 | 0.50 | 0.80 | 0.80 | 0.30 | 0.40 |
| 16 | male | 1 | 1 | 22 | 0.70 | 0.40 | 0.70 | 0.80 | 0.30 | 0.70 |
| 17 | male | 0 | 1 | 25 | 0.50 | 0.60 | 0.50 | 0.80 | 0.20 | 0.20 |
| 18 | male | 1 | 1 | 22 | 0.60 | 0.60 | 0.60 | 0.80 | 0.20 | 0.80 |
| 19 | male | 1 | 1 | 22 | 0.20 | 0.40 | 0.60 | 0.80 | 0.30 | 0.50 |
| 20 | male | 1 | 1 | 20 | 0.20 | 0.50 | 0.90 | 0.80 | 0.20 | 0.60 |
| 21 | male | 1 | 1 | 21 | 0.50 | 0.50 | 0.70 | 0.80 | 0.20 | 0.40 |
| 22 | male | 1 | 1 | 21 | 0.20 | 0.70 | 0.60 | 0.80 | 0.20 | 0.20 |
| 23 | male | 0 | 1 | 20 | 0.60 | 0.40 | 0.20 | 0.80 | 0.30 | 0.20 |
| 24 | male | 0 | 1 | 19 | 0.50 | 0.20 | 0.40 | 0.90 | 0.50 | 0.67 |
| 25 | male | 1 | 0 | 23 | 0.80 | 0.20 | 0.30 | 0.80 | 0.50 | 0.20 |
| 26 | male | 0 | 0 | 25 | 0.80 | 0.40 | 0.60 | 0.70 | 0.50 | 0.30 |
| 27 | male | 0 | 0 | 24 | 0.80 | 0.50 | 0.40 | 0.44 | 0.40 | 0.60 |
| 28 | male | 0 | 0 | 28 | 0.40 | 0.50 | 0.50 | 0.40 | 0.80 | 0.40 |
| 29 | male | 0 | 0 | 23 | 0.20 | 0.50 | 0.90 | 0.60 | 0.50 | 0.40 |
| 30 | male | 0 | 1 | 20 | 0.40 | 0.20 | 0.70 | 0.80 | 0.30 | 0.80 |
| 31 | male | 0 | 0 | 21 | 0.20 | 0.30 | 0.80 | 0.20 | 0.20 | 0.80 |
| 32 | male | 0 | 0 | 23 | 0.50 | 0.70 | 0.60 | 0.80 | 0.50 | 0.50 |
| 33 | male | 0 | 1 | 22 | 0.40 | 0.50 | 0.50 | 0.80 | 0.30 | 0.50 |
| 34 | male | 0 | 0 | 22 | 0.30 | 0.40 | 0.50 | 0.50 | 0.50 | 0.44 |
| 35 | male | 0 | 1 | 20 | 0.40 | 0.50 | 0.40 | 0.90 | 0.20 | 0.50 |
| 36 | male | 0 | 1 | 23 | 0.50 | 0.30 | 0.40 | 0.80 | 0.20 | 0.20 |
| 37 | male | 0 | 1 | 20 | 0.70 | 0.50 | 0.80 | 0.80 | 0.10 | 0.80 |
| 38 | female | 0 | 1 | 23 | 0.10 | 0.30 | 0.30 | 0.80 | 0.20 | 0.60 |
| 39 | female | 0 | 1 | 23 | 0.60 | 0.30 | 0.60 | 0.50 | 0.20 | 0.20 |
| 40 | female | 0 | 1 | 21 | 0.30 | 0.60 | 0.30 | 0.80 | 0.30 | 0.20 |
| 41 | female | 1 | 1 | 21 | 0.60 | 0.40 | 0.50 | 0.80 | 0.30 | 0.60 |
| 42 | female | 1 | 1 | 21 | 0.70 | 0.40 | 0.60 | 0.80 | 0.20 | 0.80 |
| 43 | female | 0 | 1 | 21 | 0.20 | 0.40 | 0.20 | 0.80 | 0.20 | 0.50 |
| 44 | female | 0 | 0 | 22 | 0.60 | 0.70 | 0.60 | 0.60 | 0.20 | 0.56 |
| 45 | female | 0 | 1 | 22 | 0.60 | 0.30 | 0.60 | 0.80 | 0.20 | 0.80 |
| 46 | female | 1 | 1 | 22 | 0.50 | 0.10 | 0.40 | 0.80 | 0.20 | 0.70 |
| 47 | female | 1 | 1 | 20 | 0.60 | 0.40 | 0.80 | 0.80 | 0.30 | 0.80 |
| 48 | female | 1 | 0 | 20 | 0.30 | 0.50 | 0.60 | 0.70 | 0.80 | 0.50 |
| 49 | female | 0 | 1 | 22 | 0.60 | 0.30 | 0.30 | 0.80 | 0.20 | 0.30 |
| 50 | female | 1 | 0 | 22 | 0.50 | 0.50 | 0.40 | 0.90 | 0.70 | 0.60 |
| 51 | female | 1 | 1 | 20 | 0.40 | 0.40 | 0.50 | 0.80 | 0.20 | 0.50 |
| 52 | female | 1 | 1 | 20 | 0.50 | 0.20 | 0.20 | 0.80 | 0.20 | 0.20 |
| 53 | female | 1 | 1 | 21 | 0.70 | 0.60 | 0.30 | 0.80 | 0.30 | 0.70 |
| 54 | female | 1 | 1 | 25 | 0.30 | 0.60 | 0.40 | 0.80 | 0.20 | 0.80 |
| 55 | female | 0 | 1 | 24 | 0.70 | 0.20 | 0.50 | 0.80 | 0.20 | 0.50 |
| 56 | female | 1 | 1 | 22 | 0.70 | 0.40 | 0.40 | 0.80 | 0.20 | 0.20 |
| 57 | female | 1 | 1 | 21 | 0.30 | 0.20 | 0.60 | 0.80 | 0.20 | 0.50 |
| 58 | female | 0 | 1 | 24 | 0.70 | 0.70 | 0.60 | 0.80 | 0.20 | 0.20 |
| 59 | female | 0 | 1 | 22 | 0.60 | 0.50 | 0.40 | 0.80 | 0.20 | 0.30 |
| 60 | female | 1 | 1 | 21 | 0.80 | 0.30 | 0.40 | 0.80 | 0.20 | 0.20 |
| 61 | female | 0 | 0 | 20 | 0.60 | 0.40 | 0.50 | 0.80 | 0.40 | 0.30 |
| 62 | female | 0 | 1 | 27 | 0.60 | 0.80 | 0.50 | 0.70 | 0.11 | 0.80 |
| 63 | female | 1 | 0 | 22 | 0.50 | 0.60 | 0.60 | 0.40 | 0.70 | 0.60 |
| 64 | female | 1 | 1 | 23 | 0.70 | 0.60 | 0.30 | 0.80 | 0.20 | 0.50 |
| 65 | female | 1 | 0 | 20 | 0.60 | 0.40 | 0.40 | 0.70 | 0.60 | 0.40 |
| 66 | female | 0 | 1 | 22 | 0.40 | 0.40 | 0.50 | 0.80 | 0.10 | 0.50 |
| 67 | female | 1 | 0 | 24 | 0.40 | 0.40 | 0.50 | 0.60 | 0.70 | 0.60 |
| 68 | female | 1 | 1 | 24 | 0.50 | 0.40 | 0.70 | 0.80 | 0.20 | 0.50 |
| 69 | female | 0 | 1 | 23 | 0.70 | 0.40 | 0.30 | 0.80 | 0.20 | 0.20 |
| 70 | female | 1 | 1 | 25 | 0.50 | 0.50 | 0.70 | 0.80 | 0.20 | 0.70 |
| 71 | female | 1 | 0 | 20 | 0.60 | 0.40 | 0.60 | 0.80 | 0.60 | 0.60 |
| 72 | female | 1 | 1 | 23 | 0.8 | 0.3 | 0.3 | 0.8 | 0.3 | 0.8 |

Performance on the visual search task (RTs and correct proportions of detecting target faces)

|  | (Mean RTs, 1000=1sec) | | | Correct proportions | |  |
| --- | --- | --- | --- | --- | --- | --- |
| Participant No. | Reward target | Punishment target | Zero target | Reward target | Punishment target | Zero target |
| 1 | 649.37 | 661.72 | 756.27 | 1.00 | 1.00 | 1.00 |
| 2 | 580.66 | 610.27 | 654.28 | 0.97 | 1.00 | 0.93 |
| 3 | 1074.96 | 1002.89 | 1085.70 | 0.97 | 1.00 | 1.00 |
| 4 | 805.78 | 639.59 | 752.78 | 0.63 | 0.87 | 0.66 |
| 5 | 895.47 | 790.63 | 811.36 | 0.88 | 1.00 | 1.00 |
| 6 | 763.07 | 824.15 | 897.54 | 0.84 | 0.97 | 0.74 |
| 7 | 709.21 | 699.19 | 775.61 | 0.97 | 1.00 | 0.97 |
| 8 | 807.73 | 877.00 | 877.30 | 1.00 | 0.94 | 0.91 |
| 9 | 976.30 | 969.76 | 940.07 | 0.97 | 0.94 | 0.88 |
| 10 | 928.03 | 889.68 | 1057.34 | 0.97 | 1.00 | 0.88 |
| 11 | 932.81 | 844.62 | 861.53 | 1.00 | 1.00 | 1.00 |
| 12 | 1113.85 | 1226.35 | 1199.60 | 1.00 | 1.00 | 1.00 |
| 13 | 1211.24 | 967.27 | 1113.04 | 0.88 | 0.97 | 0.87 |
| 14 | 1258.34 | 1192.63 | 1523.40 | 1.00 | 1.00 | 0.97 |
| 15 | 969.83 | 934.79 | 980.45 | 1.00 | 0.94 | 1.00 |
| 16 | 810.53 | 834.22 | 888.24 | 0.91 | 0.88 | 0.97 |
| 17 | 772.35 | 883.62 | 811.74 | 1.00 | 0.97 | 0.97 |
| 18 | 770.97 | 837.57 | 815.80 | 0.97 | 0.97 | 0.88 |
| 19 | 819.58 | 815.32 | 808.57 | 0.94 | 0.84 | 0.88 |
| 20 | 809.66 | 726.11 | 904.65 | 1.00 | 0.97 | 1.00 |
| 21 | 786.64 | 866.71 | 920.96 | 1.00 | 1.00 | 0.94 |
| 22 | 765.62 | 734.30 | 696.93 | 0.97 | 0.94 | 0.97 |
| 23 | 891.05 | 940.75 | 897.95 | 1.00 | 0.96 | 1.00 |
| 24 | 678.14 | 781.37 | 775.36 | 1.00 | 0.94 | 1.00 |
| 25 | 847.83 | 856.95 | 1002.14 | 1.00 | 0.97 | 1.00 |
| 26 | 1404.41 | 1066.52 | 1455.40 | 0.66 | 1.00 | 0.87 |
| 27 | 1560.00 | 1535.75 | 1474.58 | 0.93 | 0.84 | 0.97 |
| 28 | 792.41 | 785.03 | 768.20 | 0.93 | 0.94 | 0.94 |
| 29 | 1050.76 | 1031.61 | 949.18 | 1.00 | 1.00 | 1.00 |
| 30 | 648.39 | 801.53 | 891.97 | 0.94 | 0.94 | 0.86 |
| 31 | 933.25 | 1039.02 | 1027.80 | 1.00 | 0.97 | 1.00 |
| 32 | 911.93 | 893.67 | 902.89 | 1.00 | 0.97 | 0.97 |
| 33 | 793.78 | 746.73 | 802.50 | 1.00 | 0.97 | 0.97 |
| 34 | 1059.24 | 1091.91 | 1054.85 | 0.97 | 0.94 | 0.88 |
| 35 | 752.32 | 741.72 | 803.29 | 0.91 | 0.94 | 0.91 |
| 36 | 937.63 | 1052.35 | 1154.65 | 0.94 | 1.00 | 0.94 |
| 37 | 613.27 | 599.27 | 698.77 | 0.97 | 0.97 | 1.00 |
| 38 | 1200.71 | 1134.91 | 1167.46 | 1.00 | 0.97 | 0.97 |
| 39 | 893.18 | 832.12 | 968.53 | 0.90 | 0.97 | 0.90 |
| 40 | 910.60 | 882.14 | 879.71 | 0.72 | 0.97 | 0.84 |
| 41 | 987.79 | 1079.98 | 1057.21 | 1.00 | 0.97 | 0.94 |
| 42 | 970.12 | 1164.93 | 1020.39 | 1.00 | 0.91 | 0.97 |
| 43 | 1343.08 | 1435.37 | 1659.63 | 0.97 | 1.00 | 0.94 |
| 44 | 1061.92 | 991.60 | 1223.32 | 0.90 | 1.00 | 0.94 |
| 45 | 907.67 | 1012.07 | 982.38 | 1.00 | 1.00 | 1.00 |
| 46 | 660.32 | 693.65 | 693.12 | 1.00 | 0.97 | 0.97 |
| 47 | 1038.51 | 966.47 | 981.49 | 0.94 | 0.88 | 0.97 |
| 48 | 1090.16 | 1322.02 | 1760.43 | 0.97 | 1.00 | 0.81 |
| 49 | 957.78 | 1030.77 | 953.05 | 0.97 | 1.00 | 1.00 |
| 50 | 1455.77 | 1261.45 | 1524.82 | 0.94 | 1.00 | 0.97 |
| 51 | 914.18 | 983.01 | 980.51 | 0.84 | 0.91 | 0.88 |
| 52 | 906.66 | 866.44 | 951.62 | 0.94 | 1.00 | 0.97 |
| 53 | 858.06 | 924.93 | 927.18 | 0.91 | 0.97 | 0.91 |
| 54 | 1097.09 | 1128.80 | 1240.75 | 0.81 | 0.90 | 0.76 |
| 55 | 758.55 | 866.01 | 901.46 | 1.00 | 0.97 | 0.88 |
| 56 | 1469.49 | 1327.29 | 1350.00 | 1.00 | 0.97 | 0.97 |
| 57 | 823.61 | 856.84 | 867.86 | 0.97 | 1.00 | 1.00 |
| 58 | 1145.50 | 1067.20 | 1158.82 | 0.94 | 0.97 | 0.88 |
| 59 | 889.63 | 1011.25 | 936.99 | 0.86 | 0.97 | 0.97 |
| 60 | 1691.39 | 2072.91 | 1533.68 | 0.81 | 0.77 | 0.87 |
| 61 | 913.46 | 931.62 | 970.60 | 1.00 | 0.94 | 1.00 |
| 62 | 1120.45 | 1035.95 | 1192.15 | 1.00 | 0.97 | 0.97 |
| 63 | 710.29 | 691.87 | 817.53 | 0.63 | 0.56 | 0.59 |
| 64 | 749.11 | 739.85 | 746.36 | 0.94 | 0.97 | 0.94 |
| 65 | 1270.57 | 1491.89 | 1500.47 | 1.00 | 1.00 | 1.00 |
| 66 | 820.41 | 914.63 | 960.76 | 1.00 | 1.00 | 1.00 |
| 67 | 956.60 | 913.31 | 887.68 | 0.81 | 0.71 | 0.91 |
| 68 | 913.64 | 855.76 | 1055.19 | 0.97 | 0.97 | 0.94 |
| 69 | 1011.42 | 1043.92 | 1068.75 | 1.00 | 1.00 | 0.94 |
| 70 | 1057.59 | 1172.90 | 1058.29 | 0.97 | 0.84 | 1.00 |
| 71 | 1082.84 | 1401.04 | 1153.77 | 0.97 | 0.66 | 0.97 |
| 72 | 942.98 | 973.69 | 931.64 | 0.97 | 0.94 | 0.94 |
